# Supplementary material for: Evolutionary trajectories of snake genes and genomes revealed by comparative analyses of five-pacer viper
Source: Nat Commun. 2016 Oct 6;7:13107. doi: 10.1038/ncomms13107 (PMC5059746; doi:10.1038/ncomms13107)
Supplement: Supplementary Information — Supplementary Figures 1 - 38 and Supplementary Tables 1 - 18 [file ncomms13107-s1.pdf]

Supplementary Figures

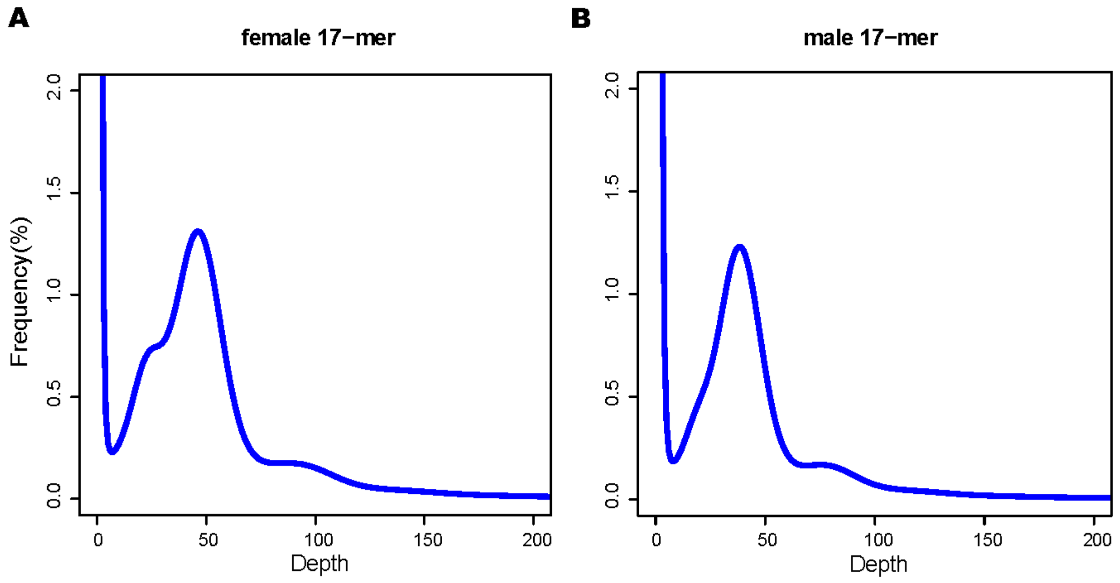

Supplementary Figure 1. K-mer estimation of the genome size of five-pacer viper. Distribution of 17-mer frequency in the used sequencing reads from female (left) and male (right) samples. The x-axis represents the sequencing depth. The Y-axis represents the proportion of a *K*-mer counts in total *K*-mer counts at a given sequencing depth. The estimated genome size is about 1.43 Gb.

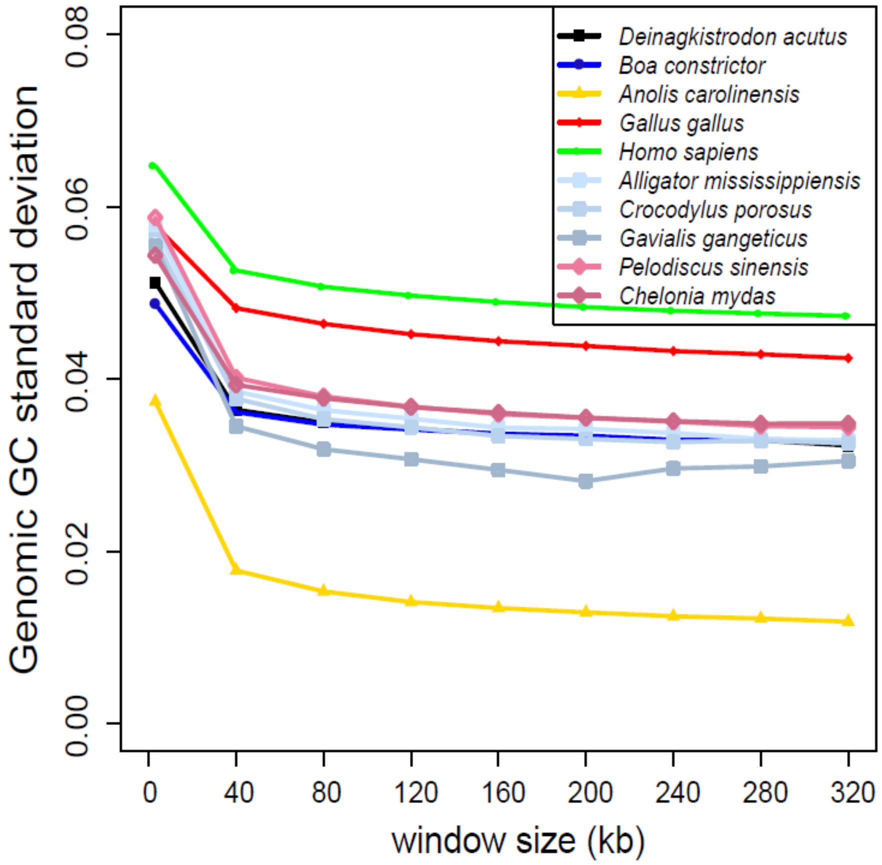

Supplementary Figure 2. GC isochore structure of different tetrapod genomes. We show the standard deviation (SD) of GC content calculated with different window size (3 kb to 320 kb) for different vertebrate genomes.

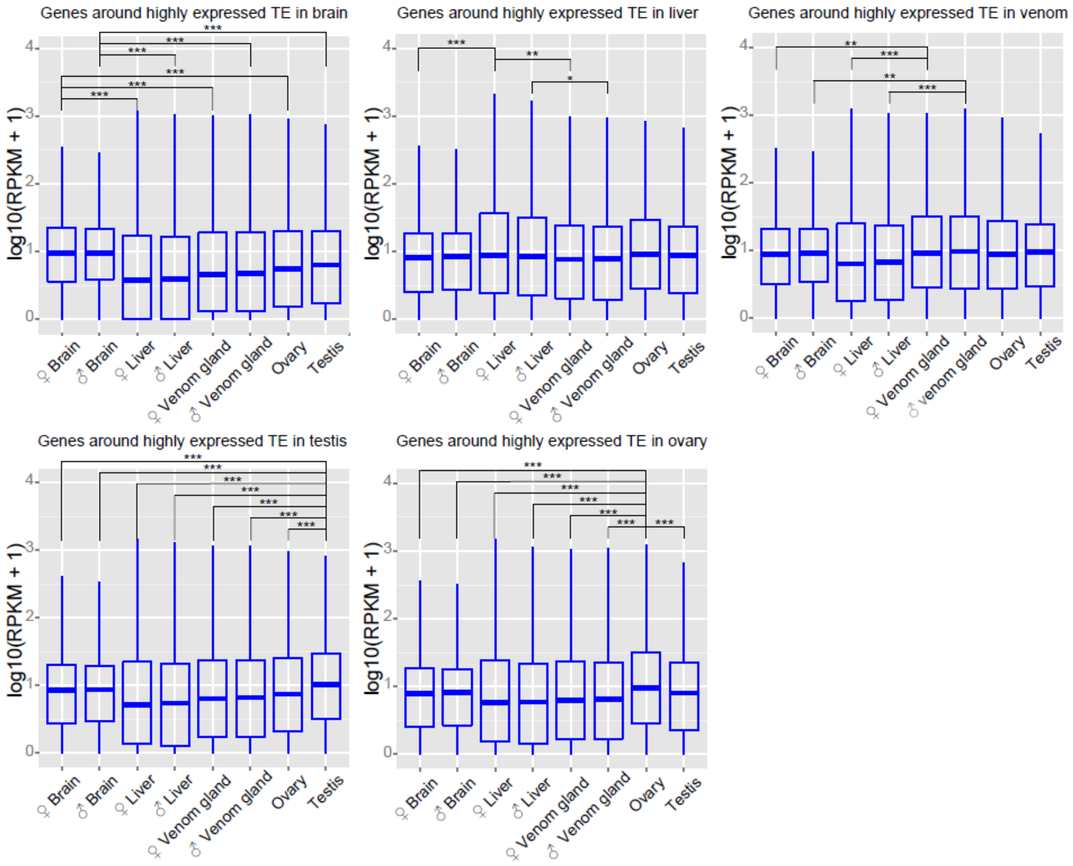

Supplementary Figure 3. Comparing expression levels of genes nearby expressed TEs in each tissue. We show expression patterns of genes around highly expressed TE (RPKM > 5) in different tissues from both sexes, including brain, liver, venom gland and gonad. We also performed comparison between the focal tissue vs. the other tissues. We show levels of significance with asterisks. *: 0.001=< *P*-value <0.01; **: 0.0001 =< *P*<0.001; ***: *P*<0.0001.

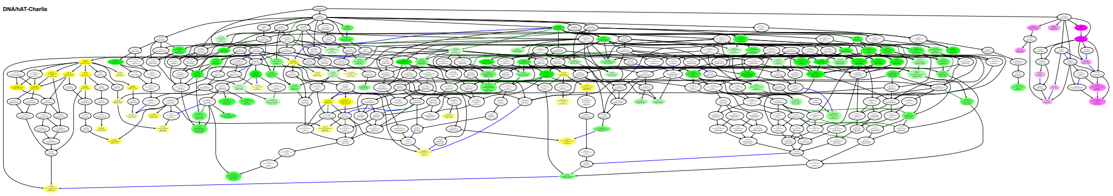

Supplementary Figure 4. GO enrichment of nearby genes of DNA/hAT-Charlie highly expressed in brain. Please zoom in for detail.

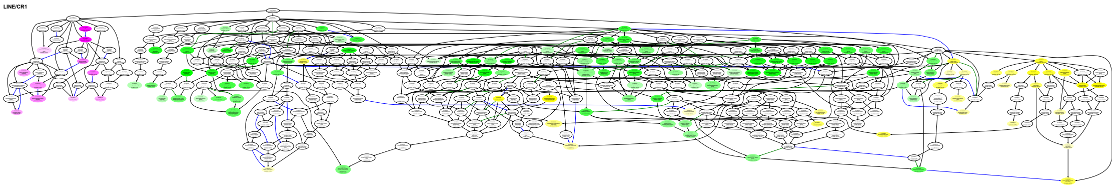

Supplementary Figure 5. GO enrichment of nearby genes of LINE/CR1 highly expressed in brain. Please zoom in for detail.

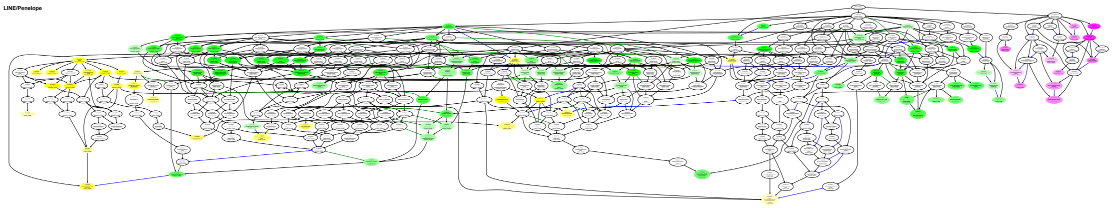

Supplementary Figure 6. GO enrichment of nearby genes of LINE/Pelenope highly expressed in brain. Please zoom in for detail.

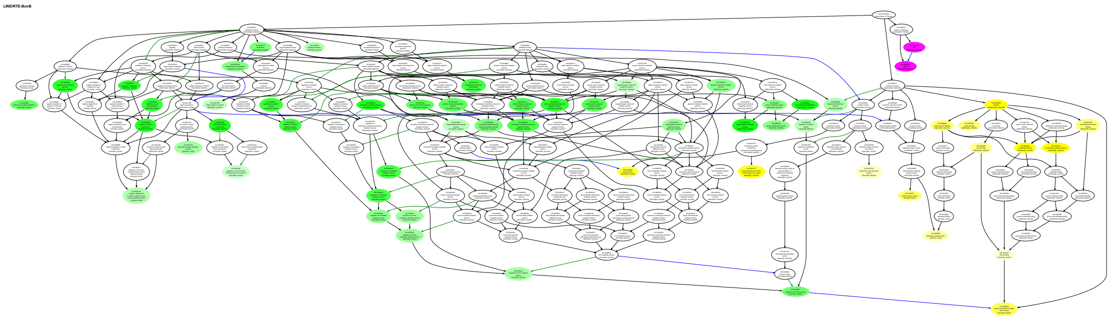

Supplementary Figure 7. GO enrichment of nearby genes of LINE/ RTE-BovB highly expressed in brain. Please zoom in for detail.

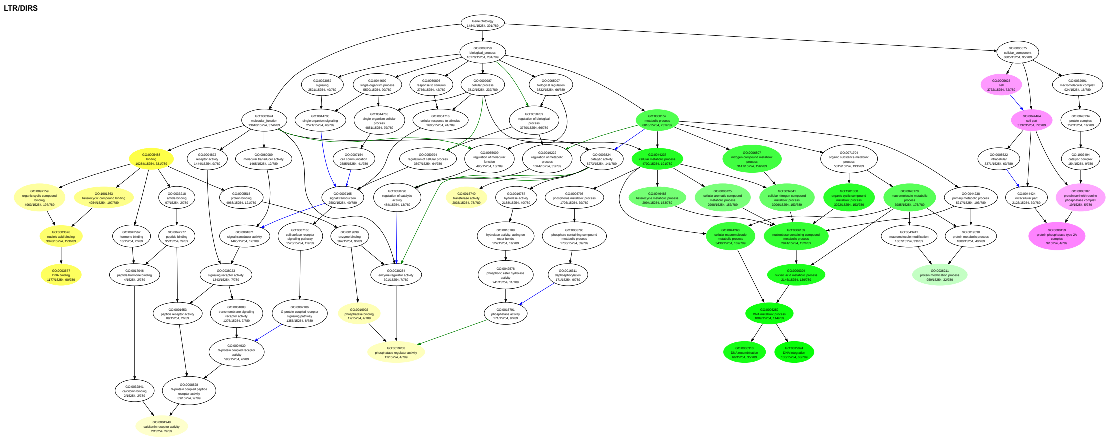

Supplementary Figure 8. GO enrichment of nearby genes of LTR/DIRS highly expressed in brain. Please zoom in for detail.

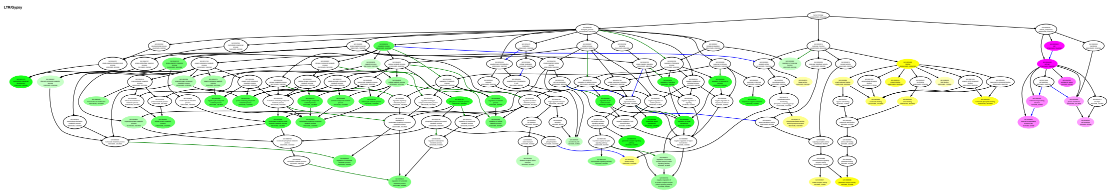

Supplementary Figure 9. GO enrichment of nearby genes of LTR/Gypsy highly expressed in brain. Please zoom in for detail.

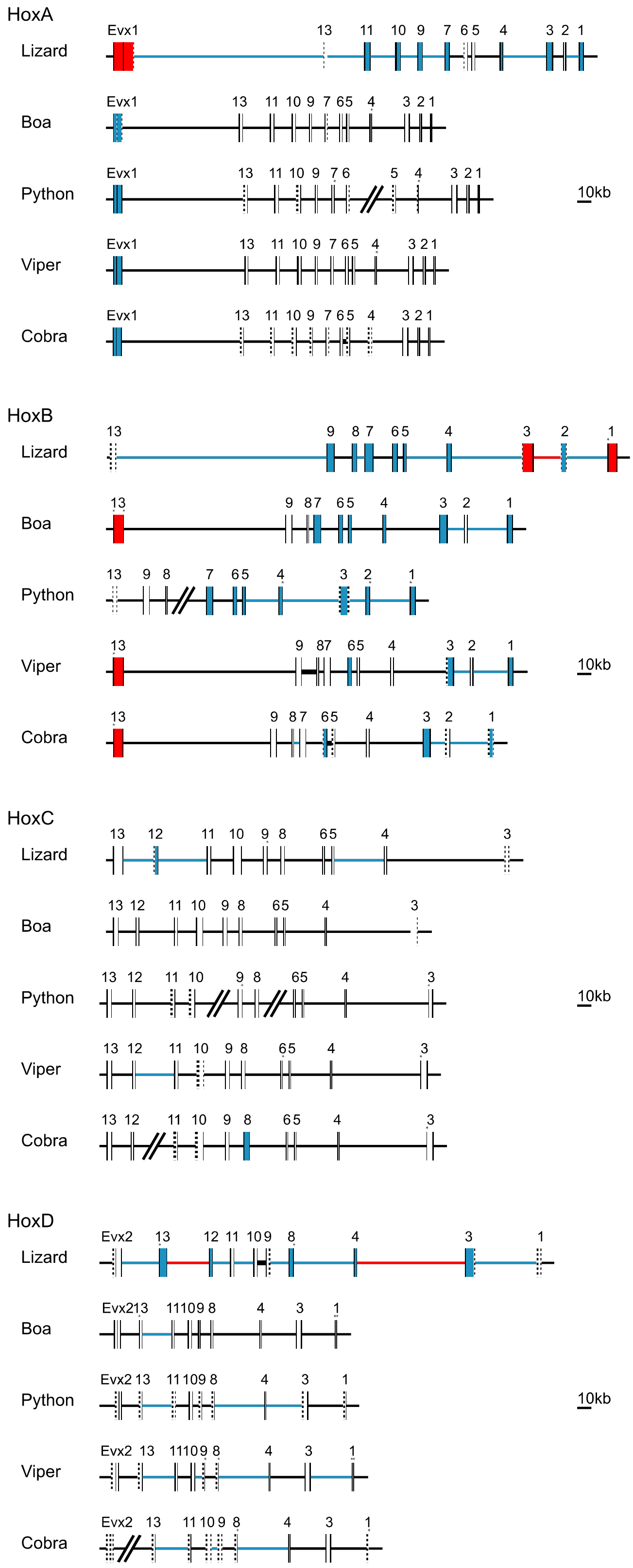

Supplementary Figure 10. Comparison of *Hox* gene structure between snakes and lizard. Schematic representation of four *Hox* clusters in anole lizard, boa,

Burmese python, five-pacer viper and king cobra. Each number from 1 to 13 denotes the specific *Hox* gene belonging to one cluster. We showed the length difference between each species vs. mouse by the colored lines (for intergenic regions) or boxes (for intronic regions): a 1.5~3 fold increase of length was shown by blue, a more than 3-fold increase was shown in red. Exons were shown by vertical lines, and dotted lines refer to exons with unknown boundaries, either due to assembly issues. Double-slashes refer to the gap between two different scaffolds.

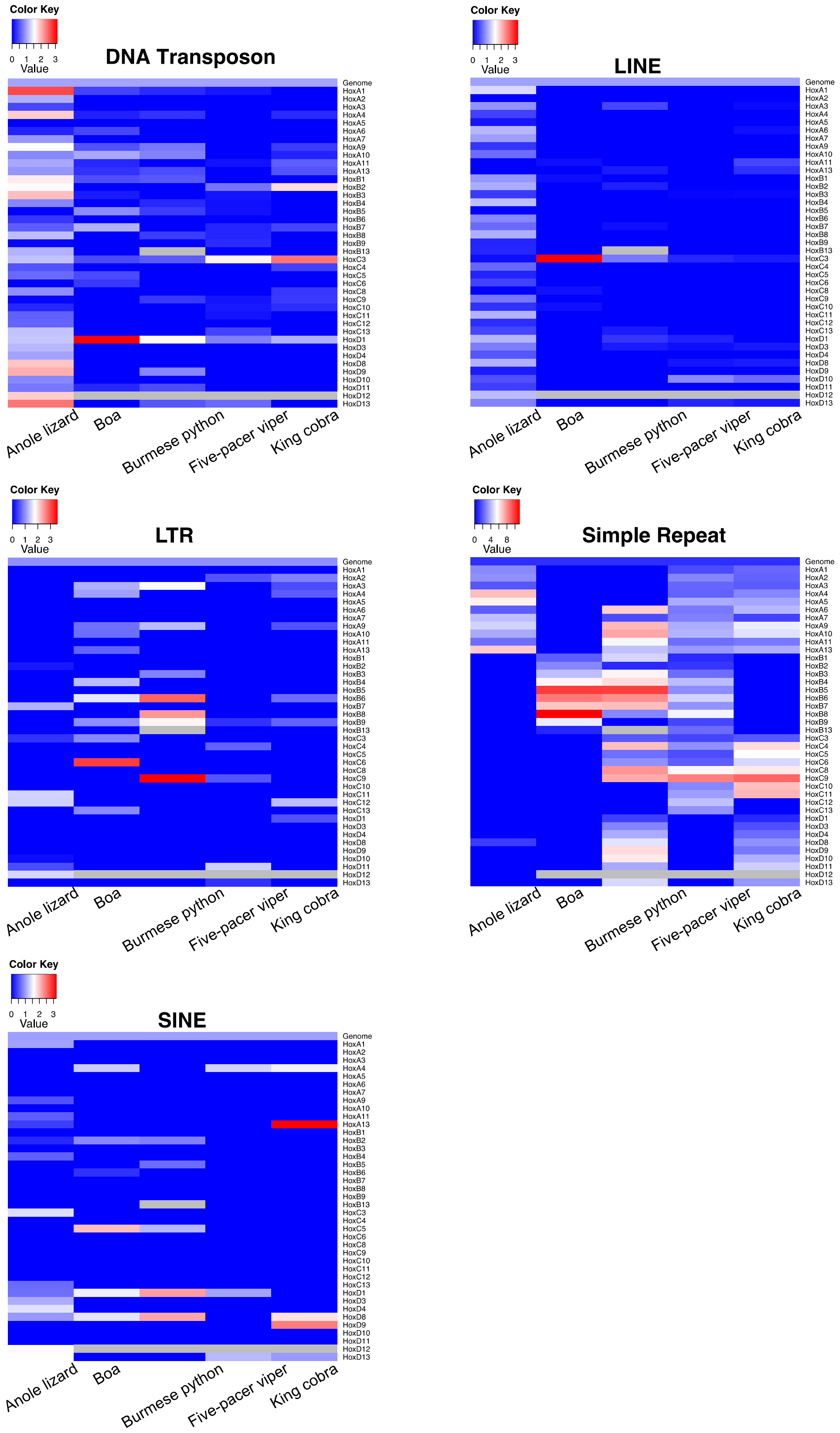

Supplementary Figure 11. Repeat accumulation at *Hox* gene clusters. Comparison of the TE and simple repeat content of *Hox* cluster genes with 5kb flanking regions between snakes and lizard. We calculated the repeat density by dividing the total length

of specific repeat sequence vs. the length of corresponding region. This density was normalized over the genome-wide repeat density and then shown by heatmap.

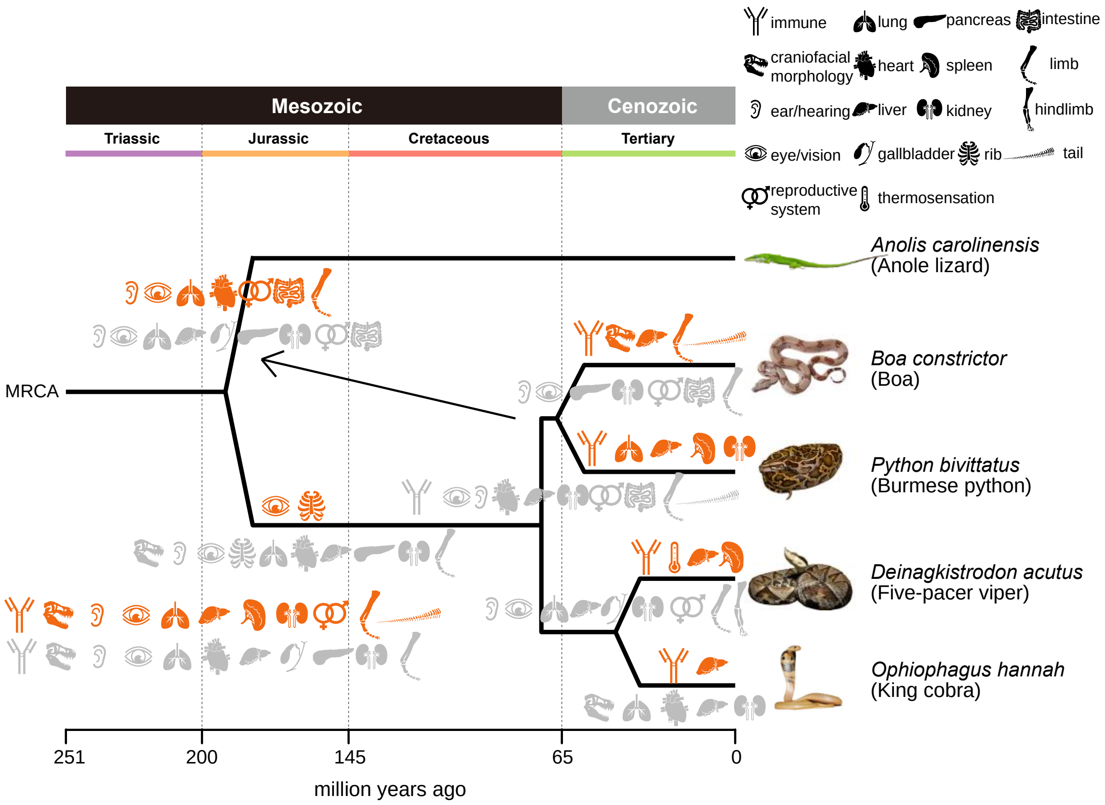

Supplementary Figure 12. Phylogenetic distribution of enriched MP terms. We identified enriched mutant phenotypes (MP) of mouse orthologs of snake genes that are undergoing lineage-specific positive selection (orange) and relaxed selective constraints (gray). And then we mapped these MP terms onto the snake phylogeny. Top-down animal photos are contributed by Mike Graziano, Sid Ewing, Camilla Bjerke, Ren-jie Wang and Zill Niazi, respectively. All rights reserved by the authors.

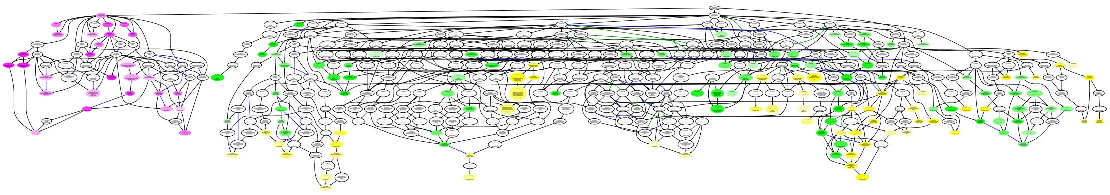

Supplementary Figure 13. Plot of GO enrichment of contracted gene families along ancestral lineage of snakes using Ontologizer. Please zoom in for detail.

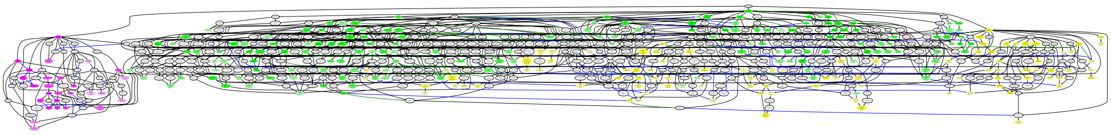

Supplementary Figure 14. Plot of GO enrichment of expanded gene families along ancestral lineage of snakes using Ontologizer. Please zoom in for detail.

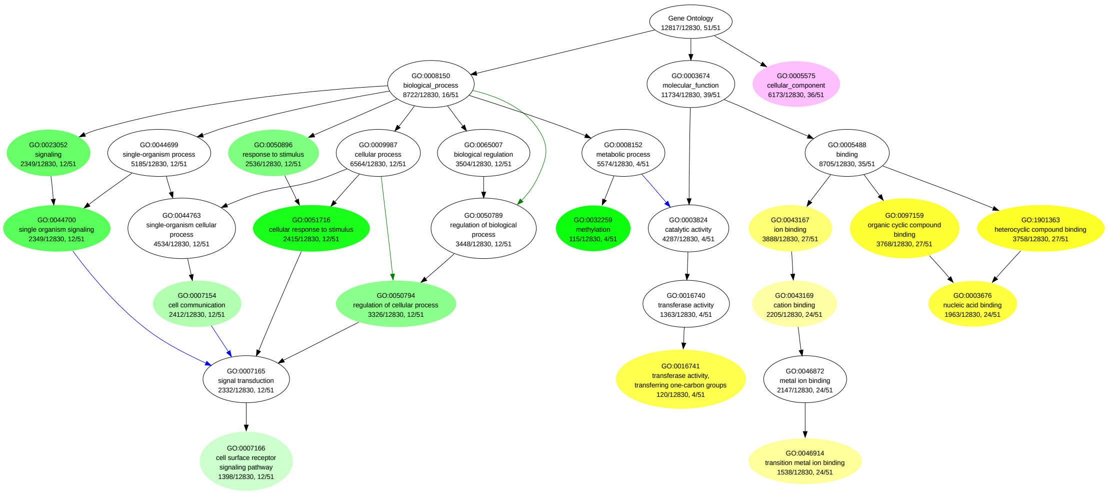

Supplementary Figure 15. Plot of GO enrichment of contracted gene families along Henophidia lineage of basal snakes using Ontologizer. Please zoom in for detail.

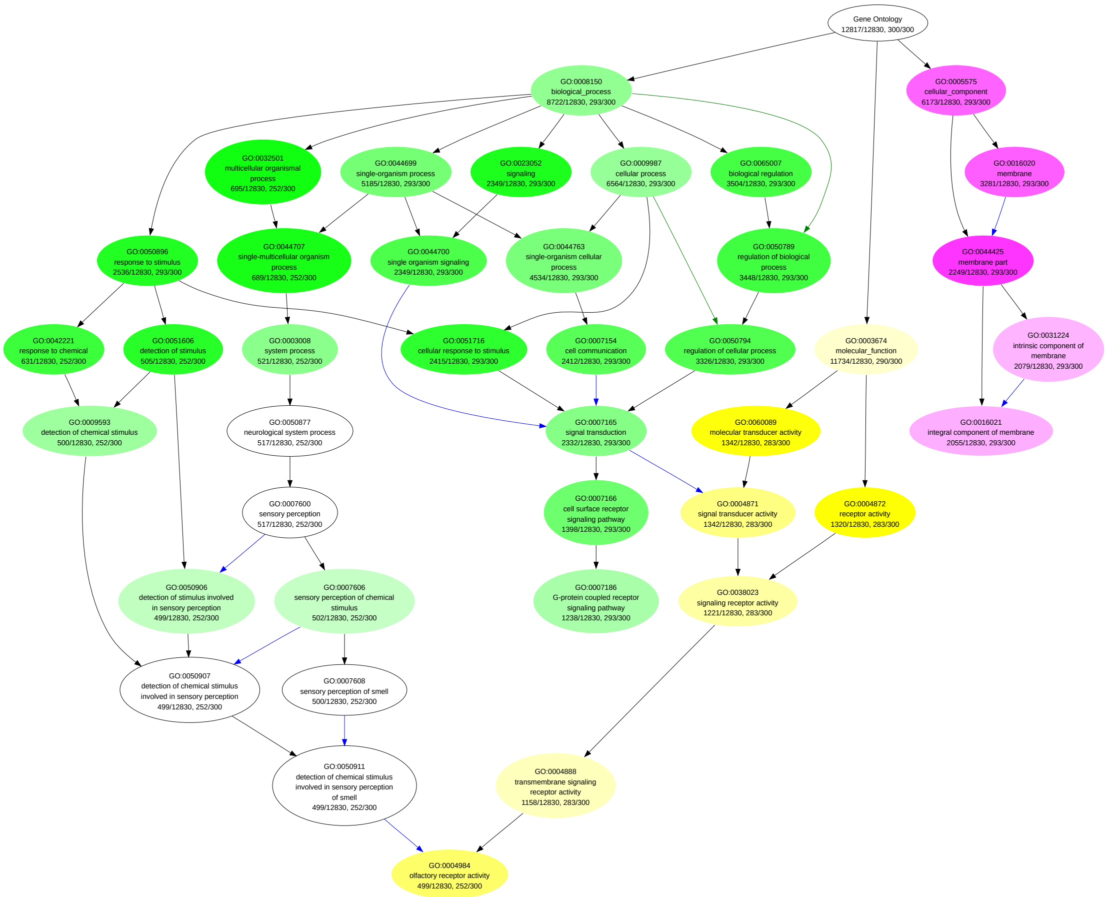

Supplementary Figure 16. Plot of GO enrichment of expanded gene families along Henophidia lineage of basal snakes using Ontologizer. Please zoom in for detail.

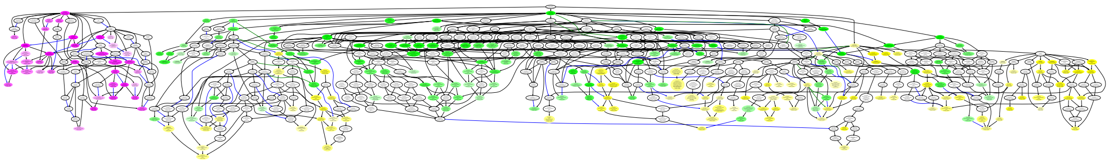

Supplementary Figure 17. Plot of GO enrichment of contracted gene families along Boa lineage using Ontologizer. Please zoom in for detail.

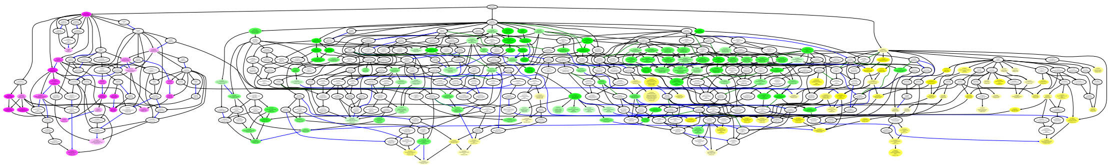

Supplementary Figure 18. Plot of GO enrichment of expanded gene families along Boa lineage using Ontologizer. Please zoom in for detail.

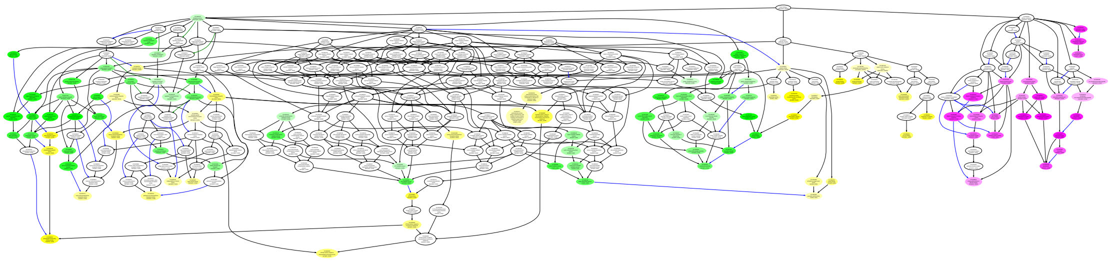

Supplementary Figure 19. Plot of GO enrichment of contracted gene families along python lineage using Ontologizer. Please zoom in for detail.

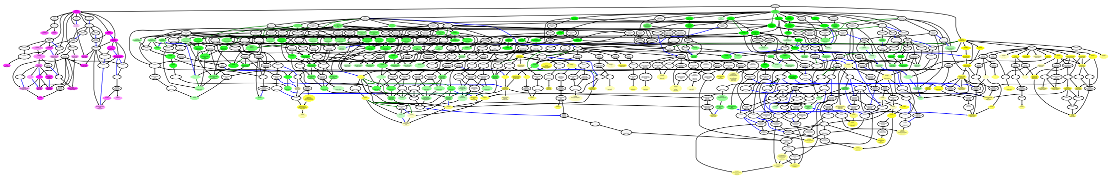

Supplementary Figure 20. Plot of GO enrichment of expanded gene families along python lineage using Ontologizer. Please zoom in for detail.

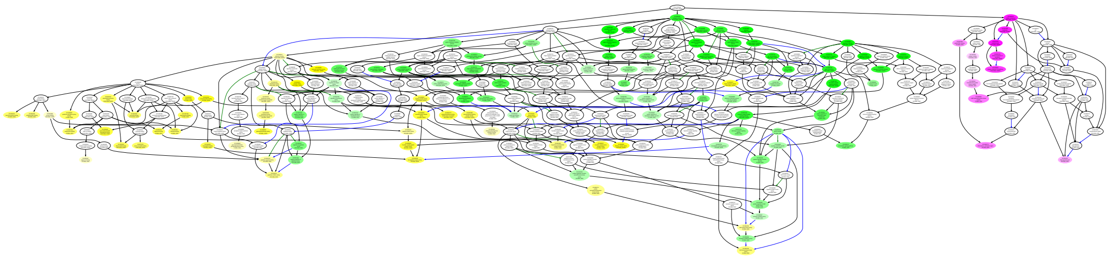

Supplementary Figure 21. Plot of GO enrichment of contracted gene families along Colubroidea lineage of advanced snakes using Ontologizer. Please zoom in for detail.

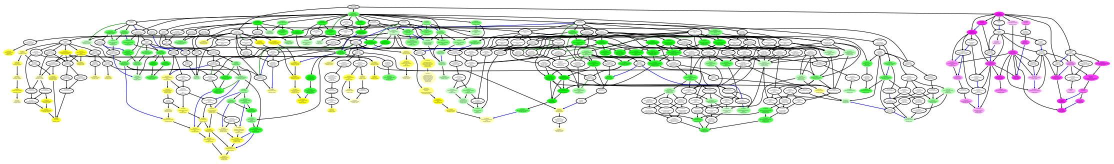

Supplementary Figure 22. Plot of GO enrichment of expanded gene families along Colubroidea lineage of advanced snakes using Ontologizer. Please zoom in for detail.

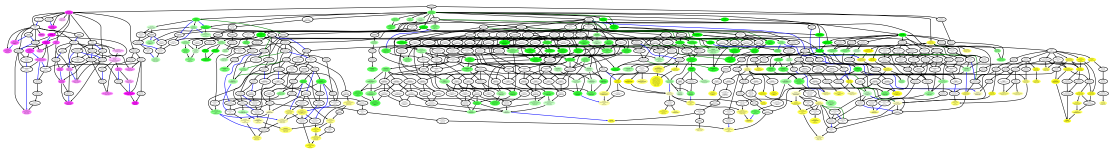

Supplementary Figure 23. Plot of GO enrichment of contracted gene families along five-pacer viper lineage using Ontologizer. Please zoom in for detail.

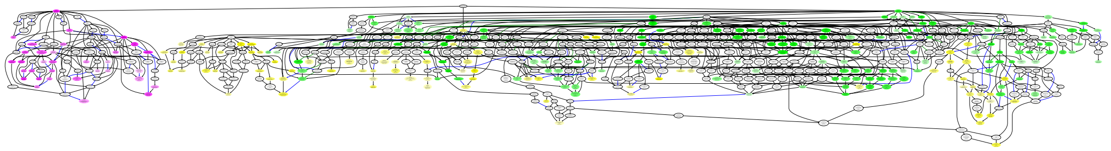

Supplementary Figure 24. Plot of GO enrichment of expanded gene families along five-pacer viper lineage using Ontologizer. Please zoom in for detail.

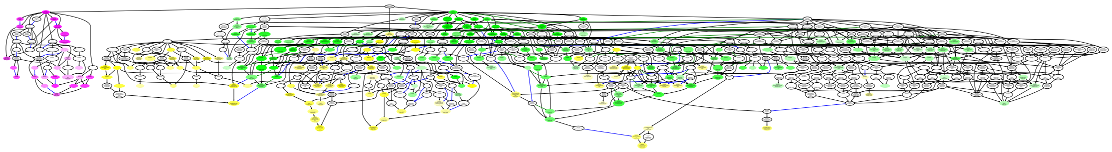

Supplementary Figure 25. Plot of GO enrichment of contracted gene families along king cobra lineage using Ontologizer. Please zoom in for detail.

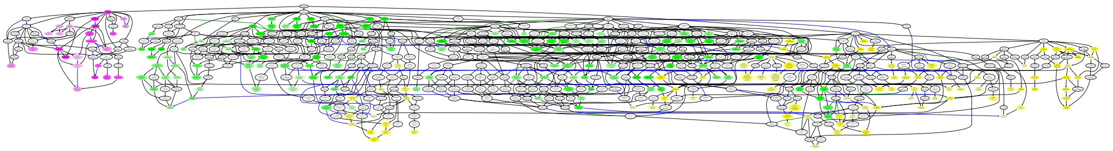

Supplementary Figure 26. Plot of GO enrichment of expanded gene families along king cobra lineage using Ontologizer. Please zoom in for detail.

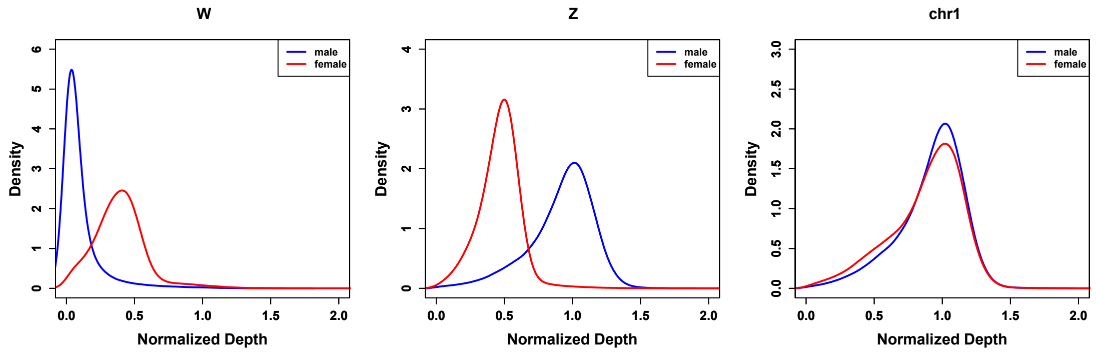

Supplementary Figure 27. Read coverage density plot of different linkage groups. For each linkage group (from left to right, chrW, chrZ, chr1), male reads were plotted in blue, and female reads in red. The identified chrW scaffolds in this work all show a female-specific read depth pattern.

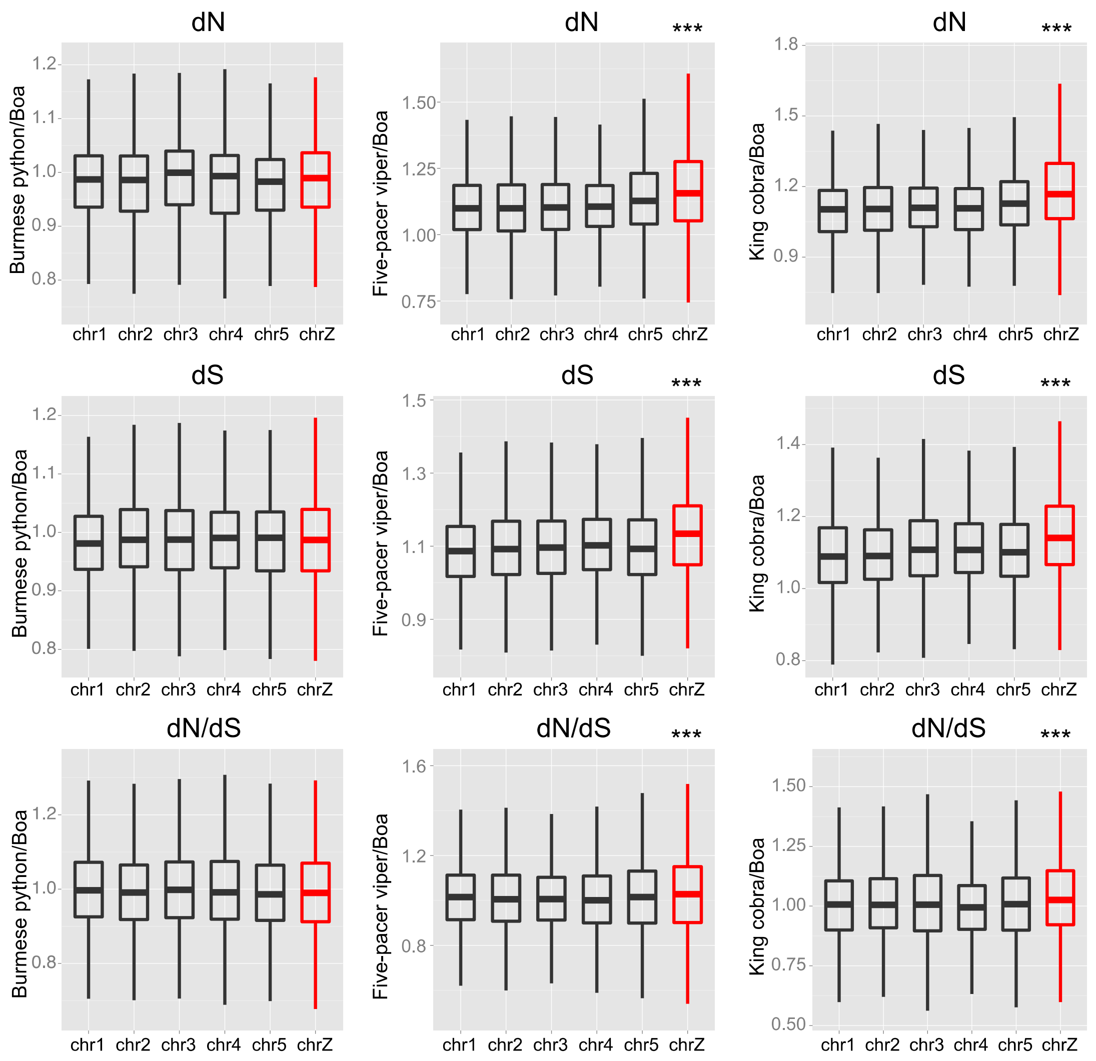

Supplementary Figure 28. Male-driven evolution effect in snakes. For each gene, we calculated the substitution rates between anole lizard and each of boa, Burmese python, five-pacer viper and king cobra at synonymous sites (dS) and non-synonymous sites (dN) divided into different chromosome sets. To detect branch-specific differences, we obtained for each gene the ratios of these evolutionary rates between the different snake species vs. boa. Since boa's homologous chromosomal region to the Z chromosomes of other advanced snakes represent the ancestral status of snake sex chromosomes, a higher relative ratios of Z-linked dS of advanced snakes vs. boa than those of autosomes indicate the male-driven evolution effect. We shown the Wilcoxon test significant differences between the Z chromosome and the autosomes (chr1-5) are marked with asterisks (***, *P*-value < 0.001).

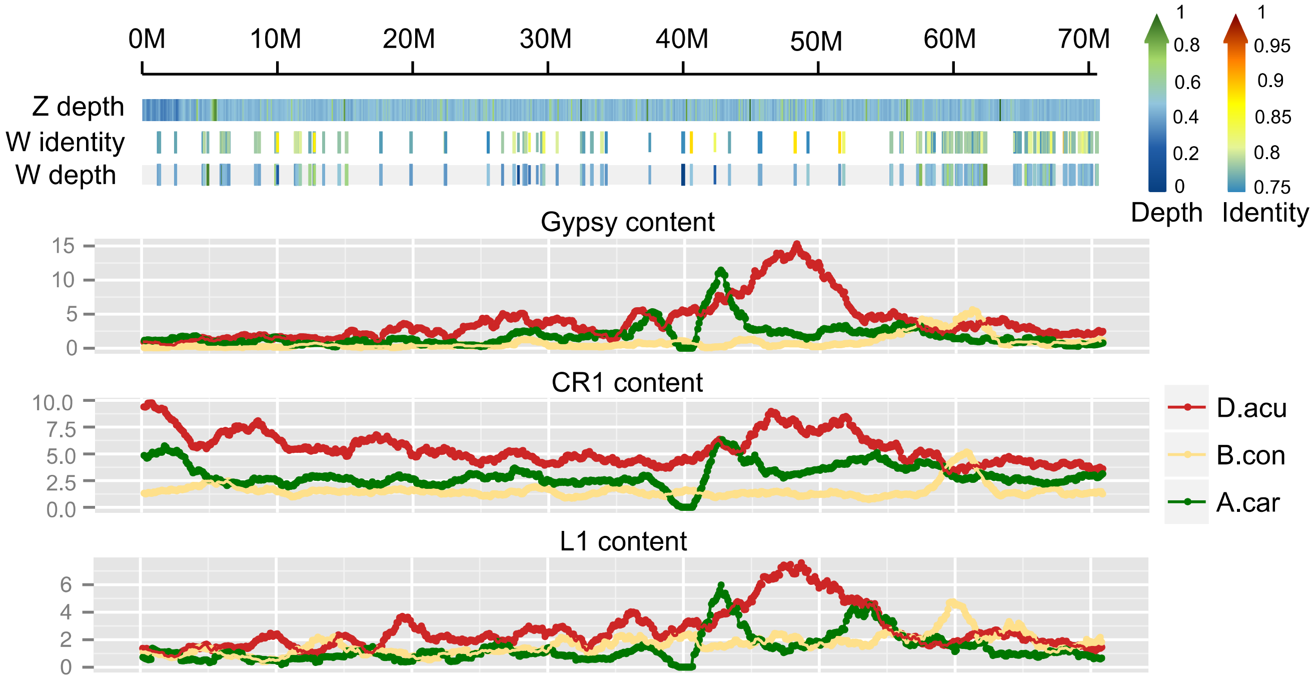

Supplementary Figure 29. Repeat accumulation along the snake sex chromosomes. Shown are comparisons of the distribution of Gypsy, CR1 and L1 content along the chromosome in anole lizard, Boa and five-pacer viper. The TE content was calculated by averaging the TE density of each sliding window of 100kb as well as the flanking 10 windows.

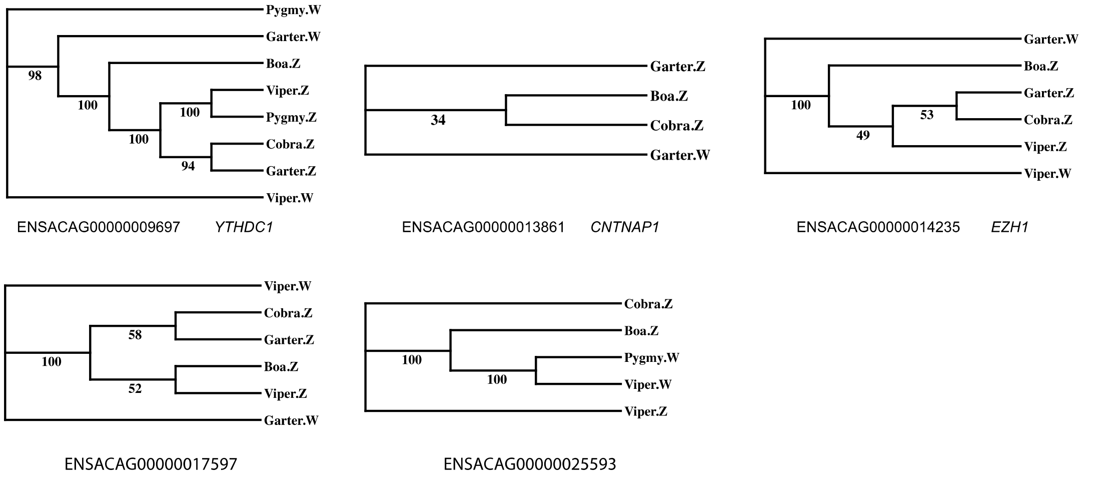

Supplementary Figure 30. Gene trees for Z and W linked gametologs in S1 region. Shown are maximum likelihood (ML) trees using coding regions of Z and W allelic sequences from multiple snake species, with the gene name under each tree and bootstrapping values at each node. Trees that show separate clustering of Z- or W-linked gametologs provide strong evidence that these genes suppressed recombination before the speciation.

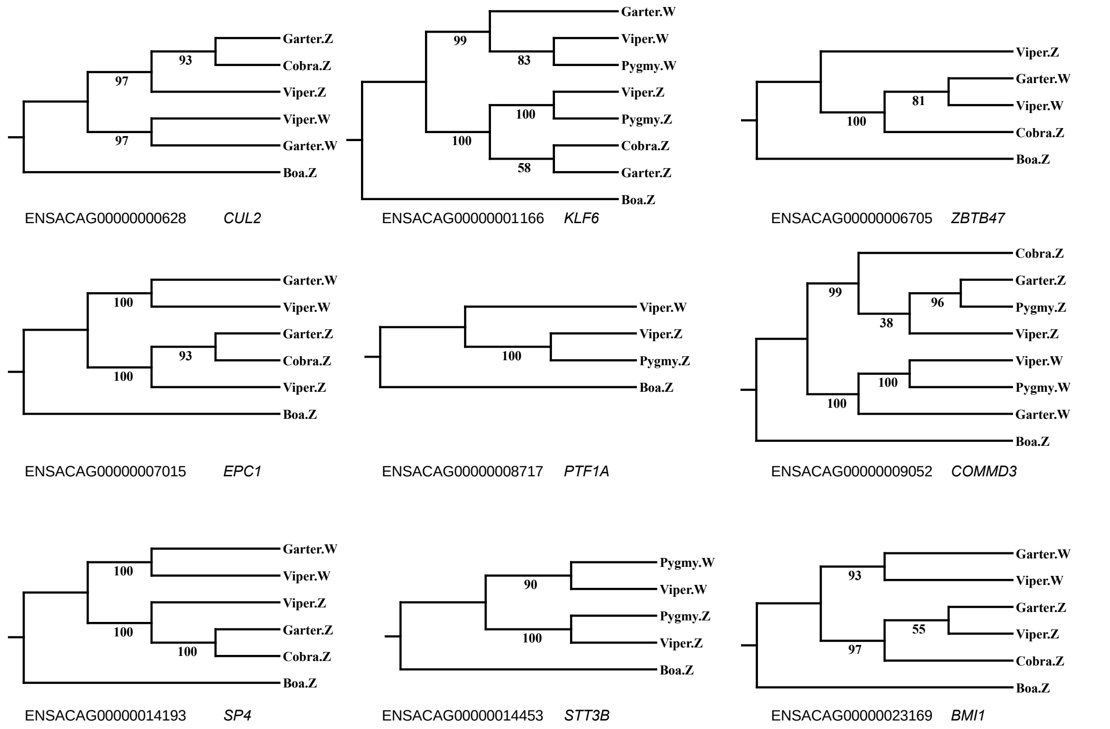

Supplementary Figure 31. Gene trees for Z and W linked gametologs in S2 region.

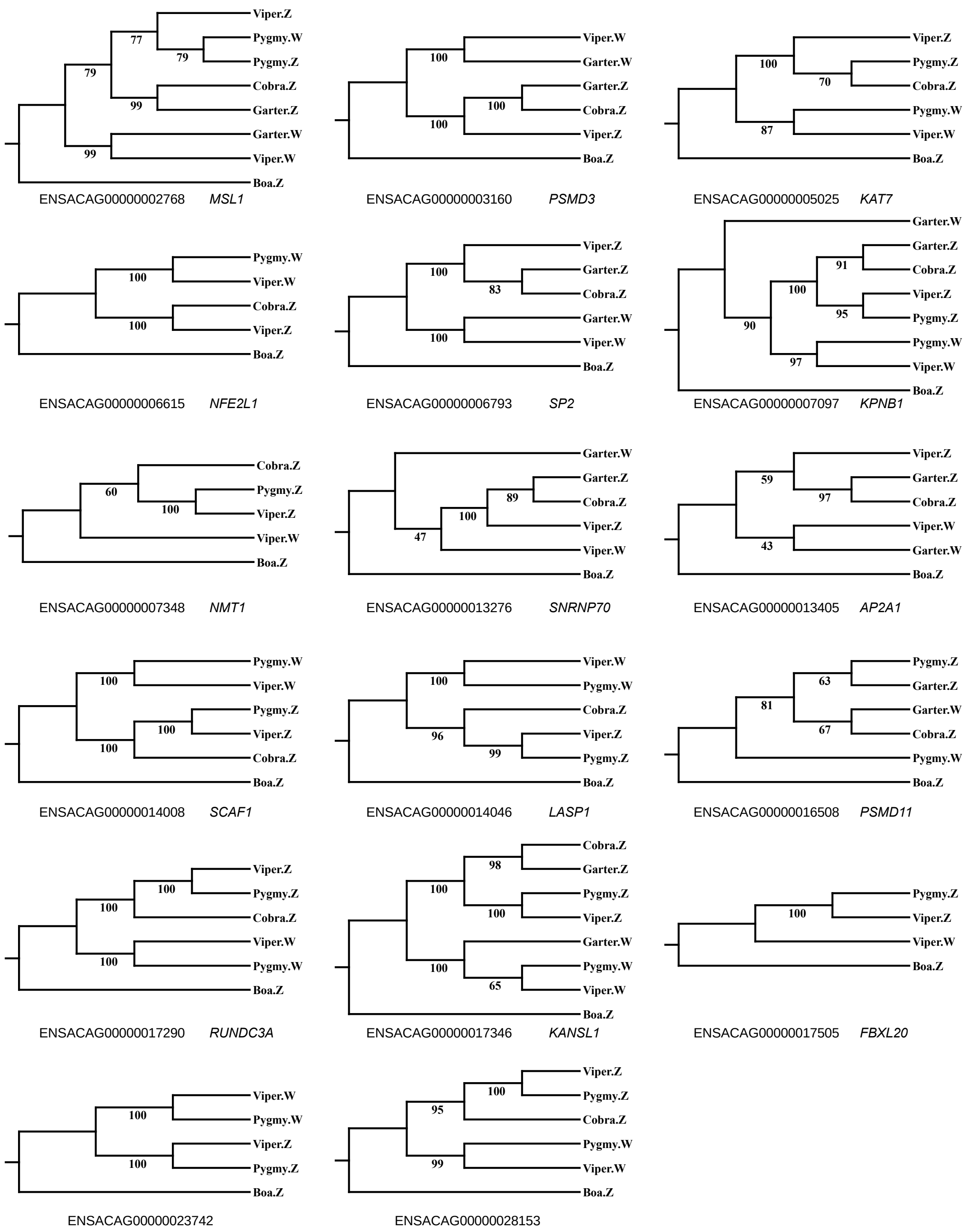

Supplementary Figure 32. Gene trees for Z and W linked gametologs in S3 region.

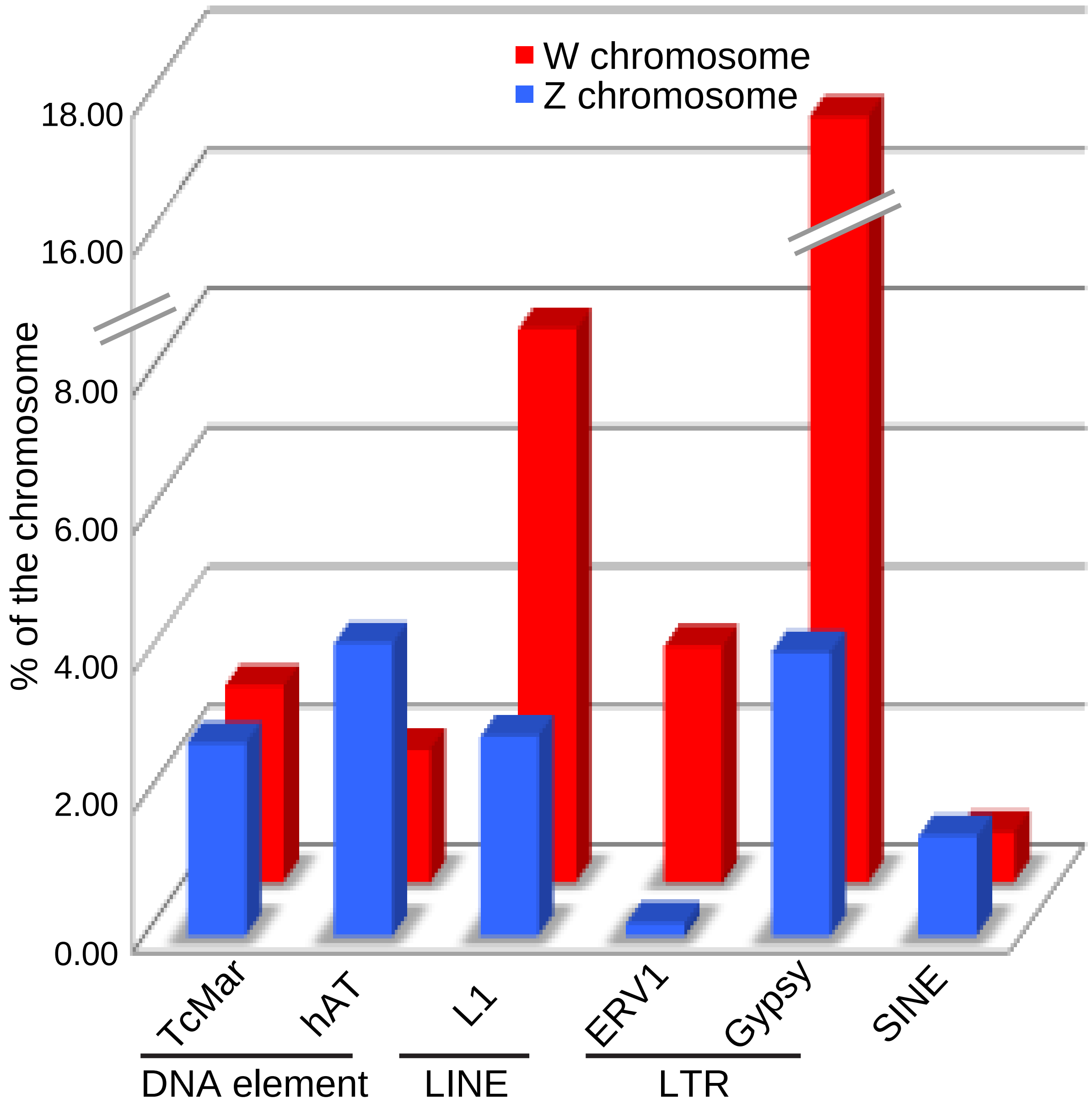

Supplementary Figure 33. Comparison of repeat content between viper chrZ and chrW. TE families were determined based on the combined annotations of Repbase, RepeatModeler, and coverage in the genome was annotated using in-house scripts.

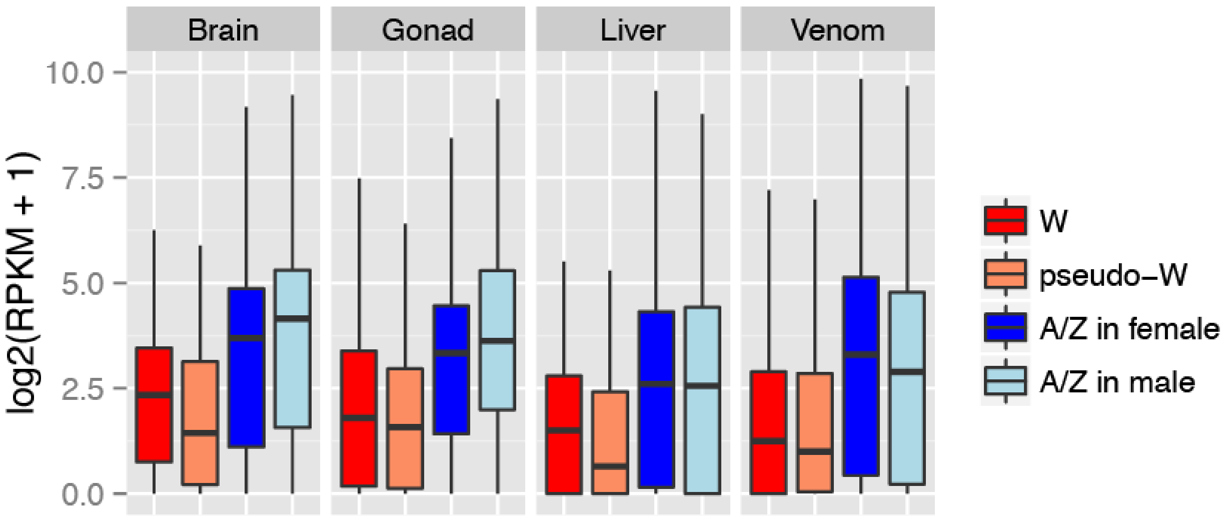

Supplementary Figure 34. Comparison of gene expression across different chromosomes. We show gene expression patterns between different chromosome sets across tissues. ‘pseudo-W’ refers to W-linked genes that have premature stop codons or frameshift mutations.

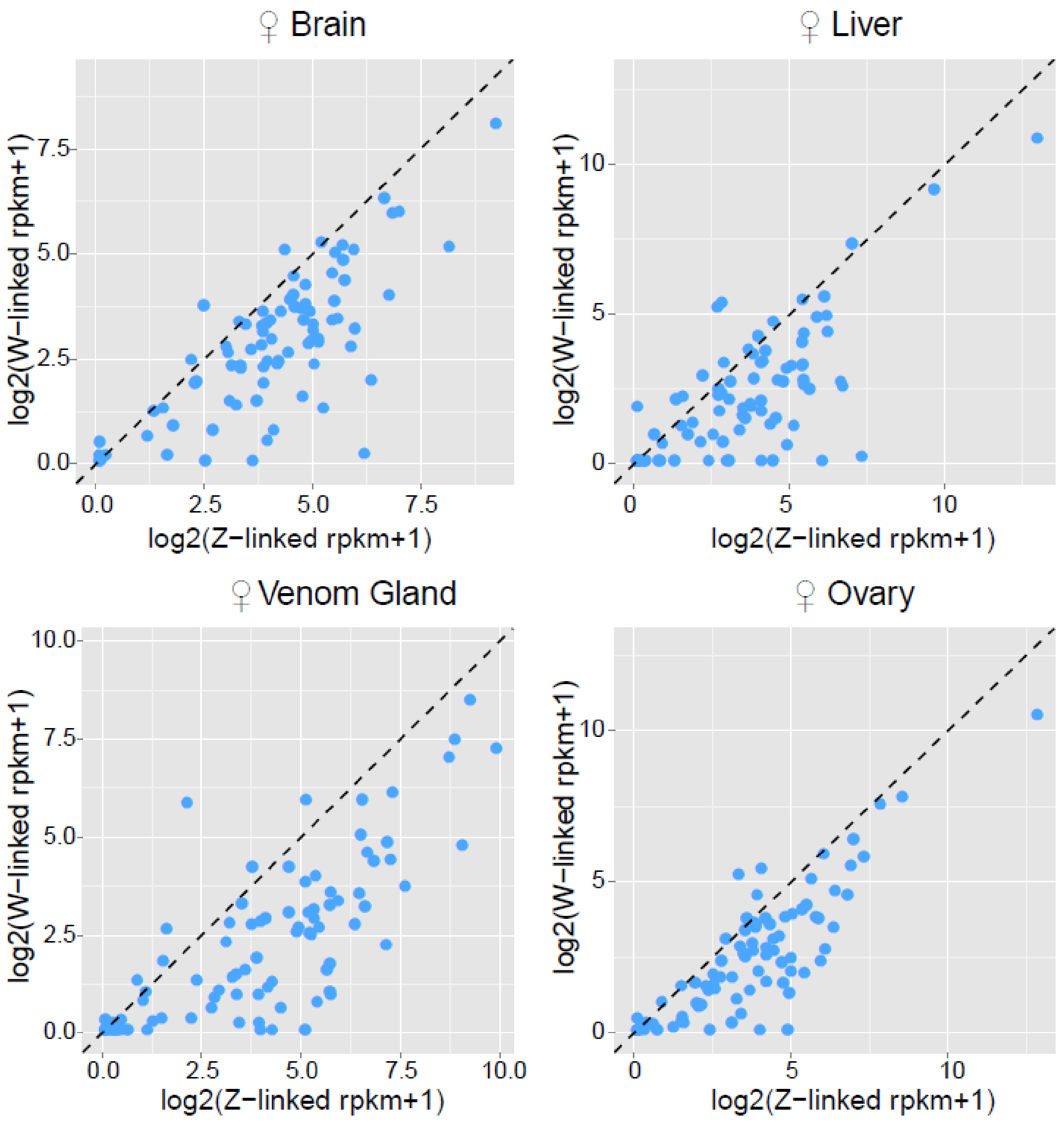

Supplementary Figure 35. Pairwise comparison of gene expression levels between homologous Z and W alleles.

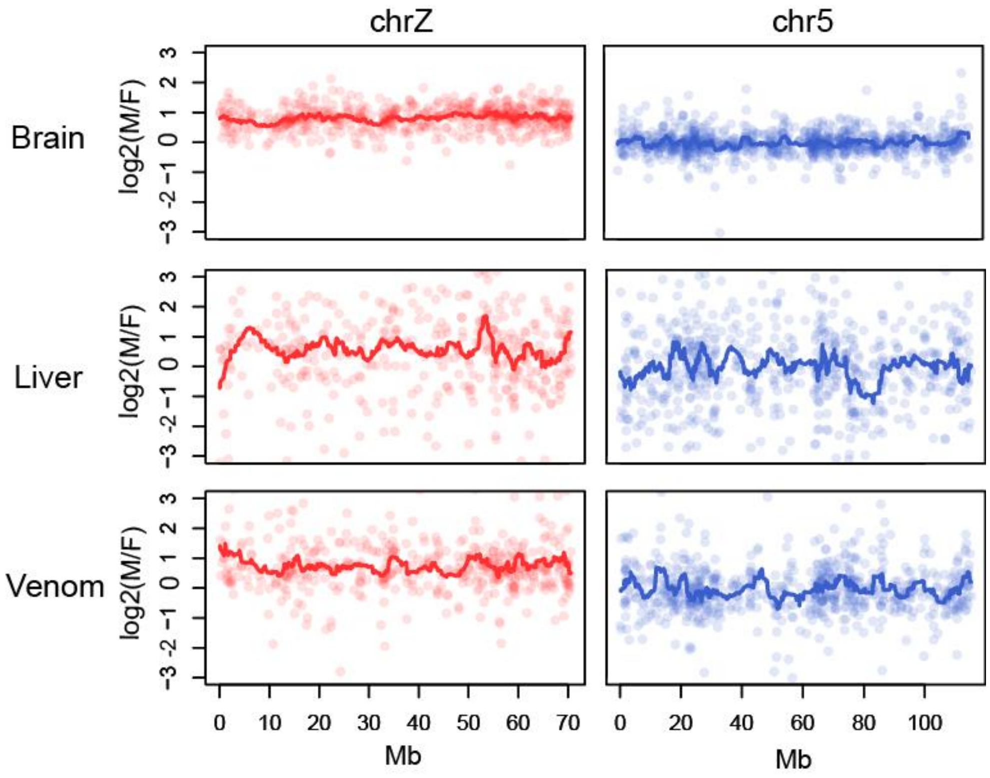

Supplementary Figure 36. Gene expression along the Z chromosome and autosome chr5 in different tissues of five-pacer viper. We show log-based male-to-female gene expression ratio along the Z chromosomes and autosome chr5. Only genes with RPKM >= 1 in both the male and female were considered. If genes are mostly non-biased, the line is expected to centered at 0. The pattern indicates five-pacer viper lacks chromosome-wide dosage compensation.

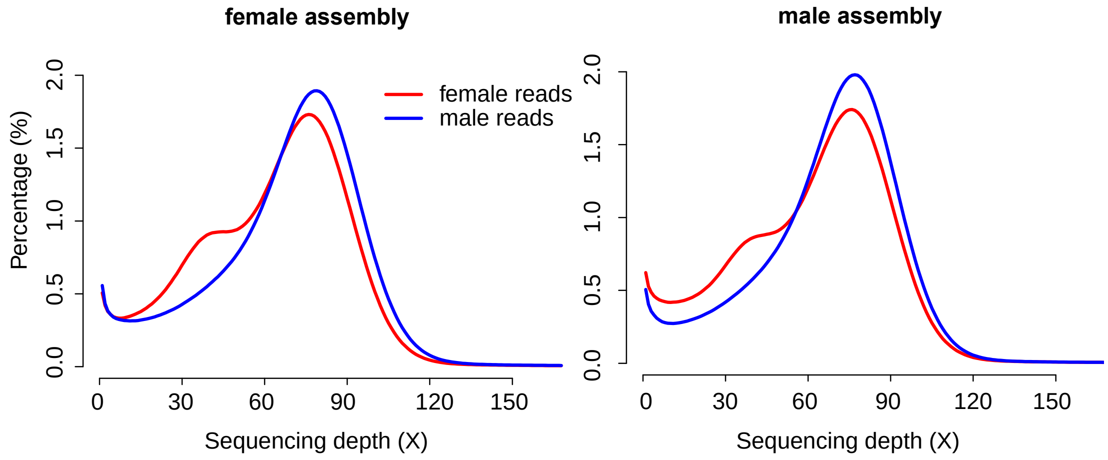

Supplementary Figure 37. Frequency distribution of sequencing depth. Distribution of sequencing depth of the assembled female (left) and male (right) genomes by reads from the female and male samples. The peak depth is 76X and 77X for the female and male reads aligned to corresponding assembly, respectively.

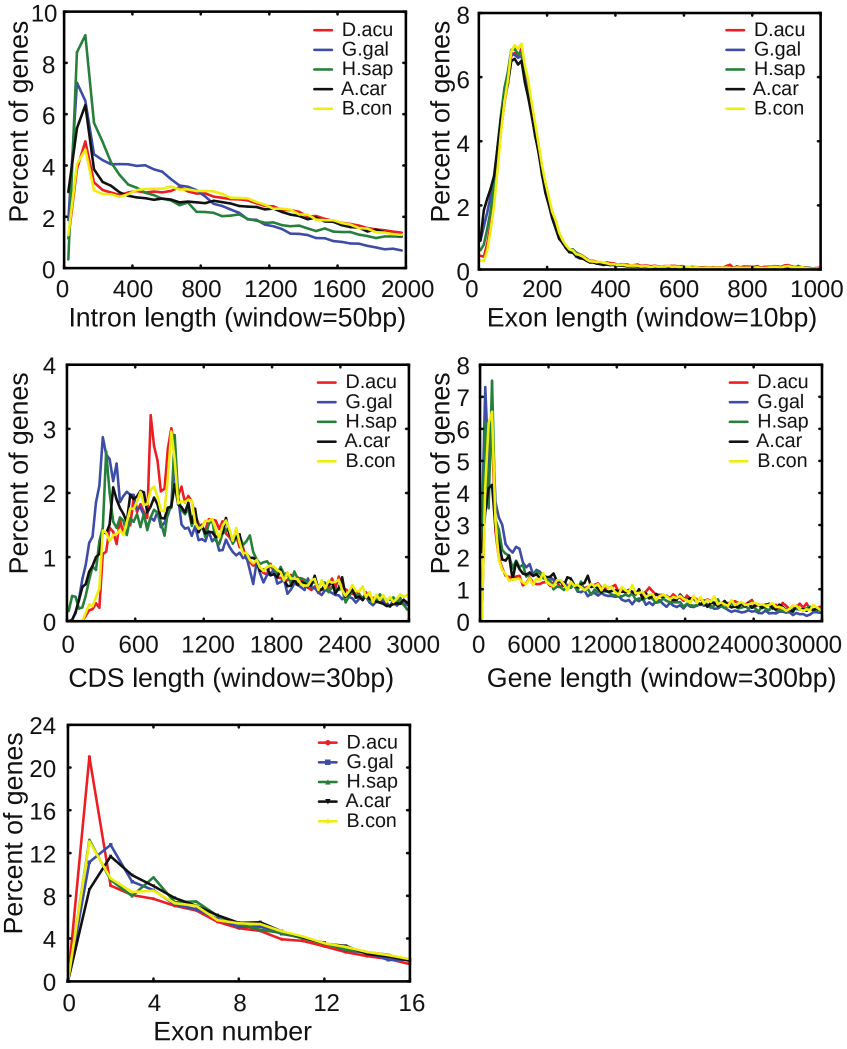

Supplementary Figure 38. Comparisons of gene parameters among the sequenced representative species. We used the published genomes of *Gallus gallus*, *Homo sapiens*, *Anolis carolinensis*, *Boa constrictor* to compare with *Deinagkistrodon acutus*, without finding any obvious differences between them and five-pacer viper in the annotated genes' length and number. This indicates the high quality of gene annotation.

Supplementary Tables

Supplementary Table 1. Statistics of five-pacer viper genome sequencing.

				Raw data		Clean data	
**Sex**	**Insert Size (bp)**	**# Library**	**Read Length (bp)**	**Total Data (Gb)**	**Sequence coverage (X) ***	**Total Data (Gb)**	**Sequence coverage (X) ***
**Female**	250	1	150	60.14	40.09	32.68	21.78
	500	1	150	61.46	40.97	39.09	26.06
	800	1	150	51.97	34.64	31.06	20.7
	2,000	2	49	42.42	28.28	23.39	15.59
	5,000	1	49	18.63	12.42	4.35	2.9
	6,000	1	49	20.19	13.46	4.67	3.11
	10,000	2	49	37.51	25.01	6.42	4.28
	20,000	2	49	26.85	17.91	2.75	1.83
	40,000	2	49	37.47	24.98	4.08	2.72
**Total**	-	13	-	356.64	237.76	148.49	98.97
**Male**	250	1	150	59.53	39.68	40.09	26.73
	500	1	100	68.03	45.36	42.79	28.52
	800	1	100	43.68	29.12	26.32	17.55
**Total**	-	3	-	171.24	114.16	109.20	72.80

*Coverage calculation was based on the estimated genome size of 1.5 Gb.

Supplementary Table 2. Statistics of 17-mer analysis.

**Sex**	**Kmer**	**Kmer number**	**Peak depth**	**Genome size (bp)**	**Used base**	**Used read number**	**Depth**
Female	17	59,943,333,424	46	1,303,115,944	69,165,384,720	576,378,206	53
Male	17	54,173,801,298	38	1,425,626,349	67,339,337,970	822,846,042	47

Supplementary Table 3. Statistics of reads of small-insert and large-insert libraries aligned to the male assembly. *PE mapped* refer to reads being mapped to the genome as read pairs, and *SE mapped* represent reads being mapped to the genome as single reads.

Type	Insert Size (bp)	Read Length (bp)	Total reads	Mapped reads	Mapped ratio (%)	PE mapped ratio (%)	Calibrated PE mapped ratio (%)*	SE mapped ratio (%)	Uniq mapped ratio (%)
Female reads	250	120	255,383,662	247,059,568	96.74	95.10	-	4.90	90.22
	500	120	319,108,900	309,556,616	97.01	93.90	-	6.10	89.34
	800	120	257,269,306	249,274,823	96.89	92.27	-	7.73	90.21
	2k	49	477,161,694	427,409,464	89.57	77.96	99.36	22.04	73.12
	5k	49	88,870,168	78,884,097	88.76	67.43	99.24	32.57	74.55
	6k	49	95,301,926	84,284,998	88.44	71.34	99.24	28.66	73.77
	10k	49	130,916,650	115,299,778	88.07	67.94	98.14	32.06	73.17
	20k	49	56,057,596	46,898,875	83.66	57.78	94.98	42.22	75.87
	40k	49	83,321,414	69,873,388	83.86	57.66	95.56	42.34	76.42
Male reads	250	135	296,994,588	289,619,546	97.52	96.08	-	3.91	92.17
	500	75;85;85	524,283,572	517,080,772	98.63	93.66	-	6.34	89.03
	800	75;85	319,511,682	313,958,573	98.26	91.50	-	8.50	88.66

* Paired-end reads from large-insert libraries without the expected insert size were excluded.

Supplementary Table 4. **Summary of the five-pacer viper genome assemblies.**

Female Sample	Contig		Scaffold	
	Size (bp)	Number	Size (bp)	Number
**N90**	5,029	61,647	231,031	977
**N80**	9,741	41,413	647,881	592
**N70**	14,673	29,372	1,043,674	405
**N60**	20,262	20,992	1,543,103	284
**N50**	26,709	14,785	2,018,329	197
**Longest**	305,908	-	12,996,529	-
**Total Size**	1,445,656,001	-	1,526,360,084	-
**Total Number (>=100b)**	-	297,390	-	183,158
**Total Number (>=2kb)**	-	85,344	-	4,414
**Gap ratio**	-	0%	-	5.29%

Male Sample	Contig		Scaffold	
	Size (bp)	Number	Size (bp)	Number
**N90**	4,457	69,307	340,274	825
**N80**	8,527	47,209	735,115	532
**N70**	12,634	33,854	1,194,490	375
**N60**	17,208	24,413	1,647,315	270
**N50**	22,424	17,322	2,122,253	192
**Longest**	219,659	-	11,788,169	-
**Total Size**	1,390,821,802	-	1,473,404,408	-
**Total Number (>=100b)**	-	287,757	-	160,256
**Total Number (>=2kb)**	-	93,050	-	2,912
**Gap ratio**	-	0%	-	5.61%

Supplementary Table 5. **Number of expressed genes of five-pacer viper.**

**Tissue**	**RPKM>0**		**RPKM>1**		**RPKM>5**	
	**Number**	**Ratio (%)**	**Number**	**Ratio (%)**	**Number**	**Ratio (%)**
♀ Brain	18545	87.50	14060	66.34	9879	46.61
♂ Brain	18407	86.85	14284	67.40	10092	47.62
♀ Liver	13428	63.36	10971	51.76	7319	34.53
♂ Liver	12910	60.91	11140	52.56	7497	35.37
♂ Venom Gland	15055	71.03	11478	54.16	8100	38.22
♀ Venom Gland	15234	71.88	11382	53.70	7855	37.06
Ovary	16858	79.54	12395	58.48	8574	40.45
Testis	17366	81.94	12780	60.30	9017	42.55
Union	19556	92.27	17134	80.84	13873	65.45

Gene expression levels were measured by RPKM (Reads Per Kilobase of gene per Million mapped reads).

Supplementary Table 6. **Number of genes and scaffold size organised into chromosomes.**

Feature	Species	Total number	# organized into chromosomes	Percent (%)	Total length (bp)	Chromosomal Length (bp)	Percent (%)
Gene	Viper	21,194	10,209	48.17	514,584,937	293,178,923	56.97
	Boa	17,392	9,154	52.63	475,414,698	293,509,235	61.74
Scaffold	Viper	160,256	629	0.39	1,473,404,408	832,463,815	56.50
	Boa	19,927	312	1.57	1,442,930,293	912,024,139	63.21

Supplementary Table 7. **Comparison of repeat content between snakes and lizard.**

Type	Anole lizard		Boa		Burmese python		Five-pacer viper		King cobra	
	Length (Mb)	% genome	Length (Mb)	% genome	Length (Mb)	% genome	Length (Mb)	% genome	Length (Mb)	% genome
**DNA**	260.26	14.47	43.62	3.02	39.24	2.73	117.35	7.96	60.75	3.81
**LINE**	274.63	15.26	188.07	13.03	185.52	12.93	203.93	13.84	196.80	12.35
**LTR**	87.32	4.85	20.82	1.44	8.30	0.58	38.15	2.59	25.08	1.57
**SINE**	105.32	5.85	38.81	2.69	26.44	1.84	26.85	1.82	33.24	2.08
**Unknown**	279.08	15.51	266.22	18.45	242.79	16.92	286.15	19.42	194.44	12.2
**Other**	36.44	2.03	28.51	1.98	26.54	1.85	46.65	3.17	41.11	2.58
**Total**	1,003.8	55.79	571.21	39.59	514.26	35.84	699.38	47.47	541.15	33.95

Supplementary Table 8. **Comparison of genome assembly quality between snakes and lizard.**

Species	Genome Size (bp)	Scaffold N50 (bp)	Total Gap Length (bp)	Length percent (%)
Anole lizard	1,799,143,587	150,641,573	97,807,040	5.44
Boa	1,442,930,293	4,505,203	55,688,379	3.86
Five-pacer viper	1,473,404,408	2,122,253	82,553,359	5.60
King cobra	1,594,074,654	241,519	215,162,447	13.50
Burmese python	1,435,034,535	213,970	50,501,725	3.52

Supplementary Table 9. Comparison of venom genes between snakes. Statistics of venom gene families in the four snakes and anole lizard genomes based on homology-based prediction. AVIT: Prokineticin; C3: complement C3; CVF: Cobra Venom Factor; CRISPs: Cysteine-Rich Secretory Proteins; Hy: Hyaluronidases; Natriuretic: Natriuretic peptide; NGF: Snake Venom Nerve Growth Factors; PLA2-2A: Snake Venom Phospholipase A2 (type IIA); SVMP: Snake Venom Metalloproteinases; TL: thrombin-like snake venom serine proteinases; LAAO: Snake Venom L-Amino Acid Oxidases; PDE: phosphodiesterases; CLPs: snake C-type lectin-like proteins; VEGF: vascular endothelin growth factor; PLA2-1B: Snake Venom Phospholipase A2 (type IB); 3FTX: The three-finger toxins; ACeH: Acetylcholinesterase;

**Venom Gene Family**	**Anole lizard**	**Boa**	**Burmese python**	**Five-pacer viper**	**King cobra**
5' nucleotidase	1	1	1	1	1
AVIT	2	2	2	2	2
Prokineticin	6	1	4	3	1
CVF	-	-	-	-	1
CRISPs	2	1	1	2	3
Crotamine	-	-	-	-	1
Cystatin	2	3	4	5	4
Hy	5	6	6	6	6
Kallikrein	7	7	1	1	3
Natriuretic	1	3	1	2	6
NGF	5	5	5	4	5
Veficolin	11	9	9	10	11
Ficolin	6	2	4	5	5
Vespryn	66	28	43	28	30
PLA2-2A	1	4	3	3	3
Waglerin	-	-	-	-	-
SVMP_I	-	2	-	1	-
SVMP_II	-	1	-	4	5
SVMP_III	1	1	2	5	4
TL	4	6	7	22	8
LAAO	4	5	6	4	3
PDE	6	6	5	5	5
CLPs	5	7	6	22	13
VEGF	4	7	7	6	5
Kunitz	86	39	49	70	53
Disintegrin_small	-	-	-	-	-
Disintegrin_dimeric	-	-	-	3	-
Disintegrin_medium	-	-	-	-	-
Disintegrin_long	-	-	-	-	-
Ohanin-like	24	12	9	14	9

factorV	5	5	6	5	5
factorX	9	11	11	11	11
Sarafotoxin	-	-	1	-	-
Waprin	5	3	3	3	4
PLA2-1B	1	1	1	1	4
3FTX	-	-	-	-	5
ACeH	22	11	12	14	16
Fasciculin	-	-	-	-	-
Total	291	189	209	262	232

Supplementary Table 10. Comparison of repeat content between snake sex chromosomes and their lizard homolog.

Type	Anole lizard chr6		Five-pacer viper chrW		Five-pacer viper chrZ	
	Length (bp)	% chr	Length (bp)	% chr	Length (bp)	% chr
**DNA**	11,737,911	14.54	1,826,436	5.55	5,747,404	7.47
**LINE**	10,617,133	13.15	5,753,245	17.48	11,492,465	14.94
**LTR**	2,624,820	3.25	7,585,819	23.05	4,249,320	5.52
**SINE**	4,761,017	5.90	236,666	0.72	1,107,788	1.44
**Unknown**	11,284,661	13.98	5,468,061	16.62	16,025,829	20.83
**Other**	1,274,093	1.58	510,501	1.55	1,679,359	2.18
**Total**	40,548,961	50.22	21,109,049	64.15	39,459,838	51.29

Supplementary Table 11. Location of W-linked putative pseudogenes.

Gene type	# Deleted/not detected			# Frameshift/premature stop codons			# Putatively functional		
	S1	S2	S3	S1	S2	S3	S1	S2	S3
Z-linked	0	0	0	21	15	3	218	447	269
W-linked	231	440	231	3	14	17	5	8	24

Supplementary Table 12. Fractions of bases covered by reads in the male assembly.

Type	Fractions of bases covered by >= N reads			
	>= 1	>= 2	>= 5	>= 10
Female reads	97.94	97.44	96.26	94.54
Male reads	99.26	98.83	97.9	96.69

Supplementary Table 13. **Characteristics of predicted protein-coding genes in the male assembly.**

**Gene set**		**Total**	**Intact ORF**	**Single exon gene**	**Gene length (bp)**	**mRNA length (bp)**	**Exons per gene**	**Exon length (bp)**	**Intron length (bp)**
**Homology based prediction**	***Anolis carolinensis***	16,861	2,587	2,049	20,351	1,523	8.37	182	2,556
	Gallus gallus	13,680	2,204	1,799	24,377	1,553	9.02	172	2,846
	Homo sapiens	14,646	2,773	1,631	28,042	1,688	9.71	174	3,024
	**Combined**	18,624	3,339	2,718	23,470	1,569	8.63	182	2,871
***De novo* predicition**		36,275	36,275	9,448	13,822	1,055	5.07	208	3,134
**Transcriptome**		41,917	26,564	23,833	6,660	884	4.21	210	1,801
**Merged**		21,194	13,368	4,089	22,010	1,583	8.39	189	2,763

Supplementary Table 14. **Number of predicted genes that can find homologs in the Ensembl library with different aligning rate cutoff.** Alignment rate was calculated by dividing the aligned length vs. the original protein length. And we required both the query and subject to satisfy our alignment cutoff. The Ensembl library consists of all proteins from *Anolis carolinensis*, *Gallus gallus*, *Homo sapiens*, *Xenopus tropicalis* and *Danio rerio*.

**Database**	**total**	**Aligning rate cutoff**		
		**>30%**	**>50%**	**>80%**
Ensembl	21,194	19,845	19,292	17,201

Supplementary Table 15. Data production and alignment statistic of RNA-Seq aligned to male genome assembly.

Sample	# Raw reads	Raw bases (Gb)	# Map reads	Map bases (Gb)	Map ratio (%)	# uniqMap reads	UniqMap ratio (%)
♀ Brain	115826248	10.42	77507446	6.98	66.92	74903732	96.64
♂ Brain	107723070	9.70	72412864	6.52	67.22	70025236	96.70
♀ Liver	117738368	11.78	77140112	7.71	65.51	73319729	95.05
♂ Liver	123319650	12.34	66982630	6.70	54.32	63883716	95.38
♂ Venom Gland	74259730	7.43	46025775	4.60	61.98	44533286	96.76
♀ Venom Gland	90752140	9.08	50146194	5.01	55.26	48034755	95.79
Ovary	72357522	7.24	50456739	5.05	69.73	48785763	96.69
Testis	96992408	8.73	69370277	6.24	71.52	67665198	97.54

Supplementary Table 16. Statistics of the identified Z-linked scaffolds.

**Z-linked scaffolds**	Size (bp)	Number
**N90**	301,572	80
**N50**	962,372	25
**Longest**	2,903,481	-
**Total Size**	76,929,177	-
**Total Number (>=100b)**	-	139
**Total Number (>=2kb)**	-	139
**Annotated gene number**	-	1,135

Supplementary Table 17. Statistics of SNPs identified in the female and male individual.

**Chromosome**	**SNP**	**Female**		**Male**	
		**Number**	**Rate (%)**	**Number**	**Rate (%)**
Autosome	Hetero-	1,373,796	0.11	1,333,210	0.10
	Homo-	1,136,245	0.09	156,910	0.01
Z	Hetero-	4,794	0.005	73,289	0.08
	Homo-	106,347	0.12	13,179	0.015
All	Hetero-	1,378,590	0.10	1,406,499	0.10
	Homo-	1,242,592	0.09	170,089	0.01

Supplementary Table 18. Statistics of identified W-linked scaffolds.

**W-linked scaffolds**	Size (bp)	Number
**N90**	10,387	123
**N50**	48,780	33
**Longest**	229,642	-
**Total Size**	32,904,513	-
**Total Number (>=100b)**	-	2,315
**Total Number (>=2kb)**	-	1,166
**Annotated gene number**	-	137
